# Evolutionary Trend of Dental Health Care Information on Chinese Social Media Platforms During 2018-2022: Retrospective Observational Study

**DOI:** 10.2196/55065

**Published:** 2025-04-10

**Authors:** Zhiyu Zhu, Zhiyun Ye, Qian Wang, Ruomei Li, Hairui Li, Weiming Guo, Zhenxia Li, Lunguo Xia, Bing Fang

**Affiliations:** 1 Department of Orthodontics Shanghai Ninth People's Hospital Shanghai Jiao Tong University School of Medicine Shanghai China; 2 College of Stomatology, Shanghai Jiao Tong University National Center for Stomatology, National Clinical Research Center for Oral Diseases Shanghai Key Laboratory of Stomatology, Shanghai Research Institute of Stomatology Shanghai China; 3 School of Computing National University of Singapore Singapore Singapore

**Keywords:** social media, dental health education, natural language processing, information quality assessment, dental care, dental hygiene, dentistry, orthodontic, health care information, retrospective study, observational study, user engagement, Chinese, dental practitioner, WeChat, health information, preventive care

## Abstract

**Background:**

Social media holds an increasingly significant position in contemporary society, wherein evolving public perspectives are mirrored by changing information. However, there remains a lack of comprehensive analysis regarding the nature and evolution of dental health care information on Chinese social media platforms (SMPs) despite extensive user engagement and voluminous content.

**Objective:**

This study aimed to probe into the nature and evolution of dental health care information on Chinese SMPs from 2018 to 2022, providing valuable insights into the evolving digital public perception of dental health for dental practitioners, investigators, and educators.

**Methods:**

This study was conducted on 3 major Chinese SMPs: Weibo, WeChat, and Zhihu. Data from March 1 to 31 in 2018, 2020, and 2022 were sampled to construct a social media original database (ODB), from which the most popular long-text posts (N=180) were selected to create an analysis database (ADB). Natural language processing (NLP) tools were used to assist tracking topic trends, and word frequencies were analyzed. The DISCERN health information quality assessment questionnaire was used for information quality evaluation.

**Results:**

The number of Weibo posts in the ODB increased approximately fourfold during the observation period, with discussion of orthodontic topics showing the fastest growth, surpassing that of general dentistry after 2020. In the ADB, the engagement of content on Weibo and Zhihu also displayed an upward trend. The overall information quality of long-text posts on the 3 platforms was moderate or low. Of the long-text posts, 143 (79.4%) were written by nonprofessionals, and 105 (58.3%) shared personal medical experiences. On Weibo and WeChat, long-text posts authored by health care professionals had higher DISCERN scores (Weibo *P*=.04; WeChat *P*=.02), but there was a negative correlation between engagement and DISCERN scores (Weibo tau-b [τb]=–0.45, *P*=.01; WeChat τb=–0.30, *P*=.02).

**Conclusions:**

There was a significant increase in the dissemination and evolution of public interest in dental health care information on Chinese social media during 2018-2022. However, the quality of the most popular long-text posts was rated as moderate or low, which may mislead patients and the public.

## Introduction

Social media usage has been extensively integrated into modern life. As of early April 2024, there were 5.1 billion social media users around the world, equating to 62.6% of the worldwide population [1]. As for Mainland China, active social media users numbered approximately 1.1 billion in January 2024, constituting 74.2% of its total population [2]. Social media provides a platform for everyone to disseminate health knowledge, demonstrate cases, and promote themselves [3]. The general public, especially patients, increasingly turns to social media to obtain health information and join communities that exchange medical experiences [4]. A survey conducted in China revealed that 71.9% of participants obtained health education through the internet, with 30.0% of them frequently seeking health information online [5]. The internet is the most important source of information for patients with cancer, and 80% of them use social media to communicate with others about their condition [6]. The content related to health on social media creates a rich repository of information, dynamically reflecting the public’s health perceptions in real time.

The growing reliance on social media for health information is a double-edged sword. On the one hand, social media offers a wealth of readily accessible information and a platform for sharing experiences, enhancing public knowledge and communication. On the other hand, studies have shown that the quality of health information on these platforms is highly variable, which may mislead the public, including patients, and potentially cause adverse outcomes [7,8]. The National Institutes of Health explicitly encourages medical professionals to share accurate health information and curb the spread of misinformation online [9].

Social media as a library for real-world studies has garnered attention from academia. Existing studies have focused on tracking topic trends, analyzing group emotions [10,11], searching for health care development directions, predicting disease spread [12], evaluating network information quality, and highlighting the harm of misinformation dissemination [13,14].

Despite the extensive user engagement and voluminous content in Chinese social media, there remains a conspicuous gap in methodical investigations into dental health care information. This gap is particularly pronounced, given China’s unique digital landscape, which is dominated by platforms such as Weibo, WeChat, and Douyin (TikTok) [8,15]. Investigating the information on these platforms could fill this void and provide Eastern insights into contemporary public perceptions and concerns regarding dental health. Although a few studies have surveyed COVID-19–related dental posts on Weibo, primarily focusing on the impact of the pandemic on patients [16,17], there is a lack of comprehensive analyses of information quality and topic trends, possibly due to limitations in research tools.

The application of artificial intelligence (AI) tools has made it possible to analyze and monitor the massive amount of information on social media [18]. Among them, natural language processing (NLP), an important branch of AI, is useful for analyzing social media content for text mining purposes [19]. Another burgeoning branch, sentiment analysis tools, can be used for public opinion analysis, such as epidemic trends [20], willingness for vaccination [10], and even presidential elections [21]. Since the emergence of Chat Generative Pretrained Transformer (ChatGPT), it has also been applied to social media research, such as popular hashtag generation algorithms [22] and the construction of lexica for online pharmacovigilance [23]. AI advancements have increased the popularity of social media research in various industries and provided guidance for their development [3,12].

This study was designed to leverage AI tools to shed light upon the changing patterns and standards of dental health care information on mainstream Chinese social media platforms (SMPs). The collected data were meticulously analyzed to identify evolving trends and assess the quality of information pertaining to dental health care. Additionally, this study probed into the determinants of audience engagement and the influence of social media information, aspiring to trace the shifting contours of public perception and the demand for dental health care. The ultimate goal was to furnish dental health care professionals with actionable insights and strategies to enhance their clinical practice and the quality of doctor-patient interactions.

## Methods

### Data Sources

This study was conducted on 3 major text-based SMPs in China: Weibo, WeChat, and Zhihu. Data from Match 1 to 31 in 2018, 2020, and 2022 were sampled. Weibo is a microblogging website. The WeChat public platform is a self-media platform based on the short-message service application WeChat. Zhihu is a knowledge question-and-answer (Q&A) community, as well as an original content platform.

### Data Extraction

To extract relevant data for this study, a social media scraping program was developed using Python’s Selenium module. The inclusion criteria specifically focused on 3 key elements: platform, time, and keywords. The time periods were divided into 3 distinct intervals: March 1-31, 2018; March 1-31, 2020; and March 1-31, 2022. The search for data extraction was conducted using a series of Chinese keywords on April 1, 2023. The translated keywords are presented in Boolean logic format in Table 1 and were divided into 3 predetermined themes (general dentistry, orthodontics, and prosthodontics) based on established classifications of subspecialties within the field of dentistry. The general dentistry section included “dental fillings,” “dental cleaning,” “tooth extraction,” “root canal treatment,” and “teeth whitening.” The orthodontics section included “orthodontics,” “teeth straightening,” “orthodontic treatment,” “braces,” “dental braces,” “get braces,” and “retainers.” The prosthodontics section included “dental crown,” “overlay,” “porcelain tooth,” “dental implant,” “implanted tooth,” “tooth veneer,” and “porcelain veneer.” The bilingual translation table is provided in Table S1 in Multimedia Appendix 1, and the translation was based on Chinese official textbooks and the Chinese edition of authoritative English textbooks [24-26]. These keywords covered a broad spectrum of nearly all commonly used Chinese expressions related to dental practices across various specialties, ensuring that the search was comprehensive and inclusive. Notably, all posts meeting the inclusion criteria were included, irrespective of authorship or topic and without manual intervention. When a post was retrieved, it was automatically assigned to the relevant theme based on the search term. For instance, posts retrieved using the keyword “dental fillings” were classified under the general dentistry theme.

**Table 1:** Keywords for searching posts (translated from Chinese).

Theme	Keywords
General dentistry	“dental fillings” OR “dental cleaning” OR “tooth extraction” OR “root canal treatment” OR “teeth whitening”
Orthodontics	“orthodontics” OR “teeth straightening” OR “orthodontic treatment” OR “braces” OR “dental braces” OR “get brace” OR “retainer”
Prosthodontics	“dental crown” OR “overlay” OR “porcelain tooth” OR “dental implant” OR “implanted tooth” OR “tooth veneer” OR “porcelain veneer”

Weibo’s open application programming interface (API) allowed access to all content that met the inclusion criteria. In contrast, for WeChat and Zhihu, we could rely only on the built-in search functions, which do not provide access to all the data. Zhihu required searches based on time conditions within the website. For the WeChat public platform, Sogou’s WeChat search was conducted to retrieve the top 10 pages of posts based on the website’s own sorting logic, representing the content that people were most likely exposed to.

The exclusion criteria were primarily based on 4 aspects. “Duplicated” content referred to posts that were entirely identical due to duplicate publication, plagiarism, or other similar reasons. Only the earliest published post was retained, and the others were removed. “Irrelevant” content referred to posts in which dental-related keywords were mentioned only briefly, while the main content focused on unrelated subjects. “Unavailable” content referred to posts where the title or abstract was accessible but the full text was no longer available, possibly due to voluntary removal or deletion by the platform. Finally, “meaningless” content included posts that were composed of incoherent or garbled text consisting of nonsensical strings of words or symbols without any thematic relevance. Upon data collection, the content from the 3 SMPs was rigorously screened according to the exclusion criteria. Posts that could potentially disrupt the integrity of the subsequent analysis were removed, and the remaining posts were included to construct a social media original database (ODB).

### Database Construction

Following data collection and screening based on the inclusion and exclusion criteria, the ODB was constructed. Posts with 600 characters or more were defined as “long-text posts.” Previous studies suggest that long-text posts tend to provide more comprehensive information and exhibit higher engagement levels [27]. The top 20 long-text posts with the highest level of popularity each month on the three platforms were selected to establish an analysis database (ADB) for lexical analysis with NLP tools and information quality assessment.

### Evaluation Strategy

Preliminary analysis of the ODB data involved capturing author information and popularity indicators for each platform, including the number of likes or reads. The metric for popularity varied by platform. For Weibo and Zhihu, the number of likes was used. For WeChat, the number of reads was used, as WeChat users tend to use “like” functions less frequently. The top 20 long-text posts with the highest levels of popularity during each observation period were selected from each platform, totaling 180 posts constituting the ADB for text mining and information quality evaluation. The classification of author account types followed subsequently and was based primarily on usernames. Specifically, accounts with names containing medical-related terms, such as “doctor,” “dentist,” “hospital,” or “clinic,” were classified as health care professionals, whereas those without such identifiers were categorized as non–health care professionals.

Text mining analysis and information quality evaluation were conducted on the ADB. NLP tools were used for text mining analysis. Using the Jieba segmentation tool, the first step involved cleaning the text data by removing punctuation, converting all text to lowercase, and eliminating common Chinese stop words, such as “of.” The cleaned text was then tokenized into individual words, or tokens, to facilitate the analysis of word frequency across the entire ADB. Python’s Counter module was used to compile a frequency distribution of the keywords. The most frequently mentioned words were visualized as word clouds, and the frequencies of the top 30 words for each period or platform were visualized as heatmaps. The font size of the word cloud and the color depth of the heatmap represented the frequency of word occurrence, providing a clear representation of the prevalent topics and their temporal variation. For information quality evaluation, the DISCERN questionnaire (Multimedia Appendix 2), which is widely used in research on the quality of health material information online [28,29], was used to assess the reliability of health material and the quality of information for treatment plan selection. The DISCERN score was rated on a 5-point Likert scale, with 1 indicating low quality, 5 indicating high quality, and 3 indicating moderate quality [30]. To ensure the objectivity of evaluation, all posts were anonymized during the review and quality assessment process. The determination of account type was conducted separately from the quality scoring, ensuring that the account type did not influence the quality assessment. Given the greater complexity of the DISCERN information quality assessment, this work was conducted by 2 practicing dental professionals following the Cohen kappa consistency test. Among the 180 long-text posts in the ADB, 18 (10%) were randomly selected for independent assessment by 2 evaluators, resulting in a Cohen kappa coefficient of 0.84, indicating reliable consistency between the results of the 2 evaluators.

### Statistical Analysis

Statistical analysis was performed using SPSS software version 25 (IBM Corp). Descriptive results are presented as the median (25th-75th percentile) for quantitative data. The Shapiro-Wilks test and the Levene test were performed to determine the normality and homogeneity of variance, respectively. The Mann-Whitney U test and the Kruskal-Wallis test, followed by Bonferroni correction, were used to compare nonparametric data among the groups. The Kendall tau-b (τb) correlation coefficient was calculated to evaluate the potential relationships between the parameters. The significance level was set at *P*<.05.

### Ethical Considerations

This study did not seek ethical approval, as it exclusively analyzed publicly available data from SMPs, which were voluntarily shared by users in the public domain. All data has been deidentified; account information collected was used solely for research analysis, and relevant results are presented in aggregate to ensure that no personally identifiable information is disclosed.

## Results

### Post Details

**Figure 1:**
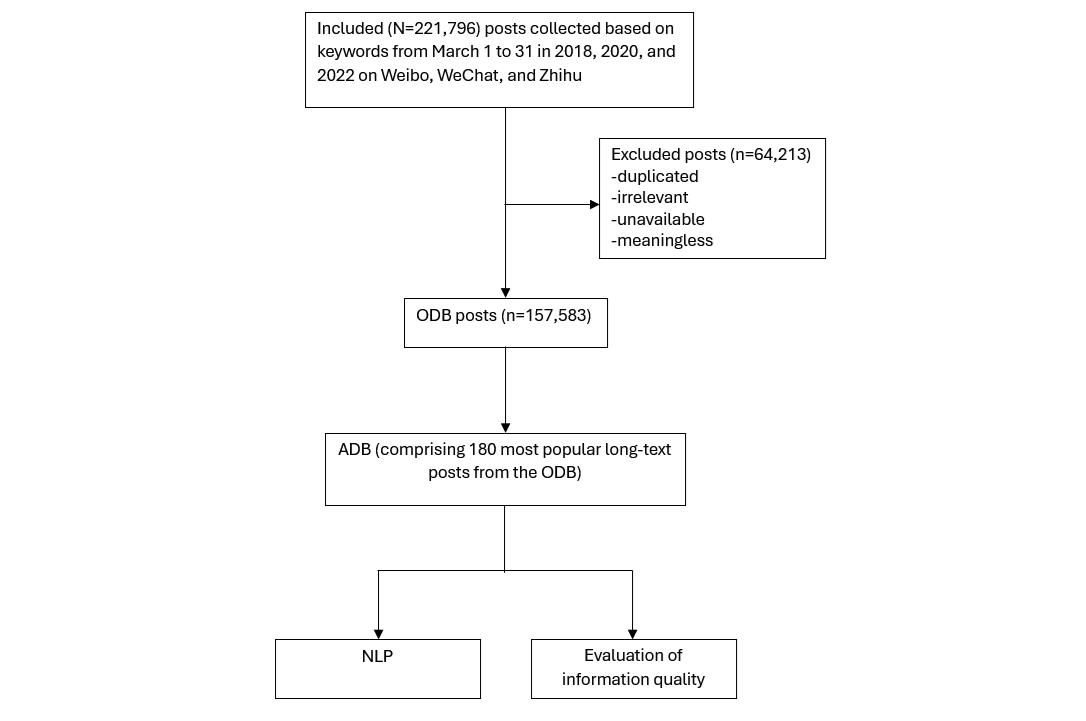
Overview of data-processing flowchart. ADB: analysis database; NLP: natural language processing; ODB: original database.

**Figure 2:**
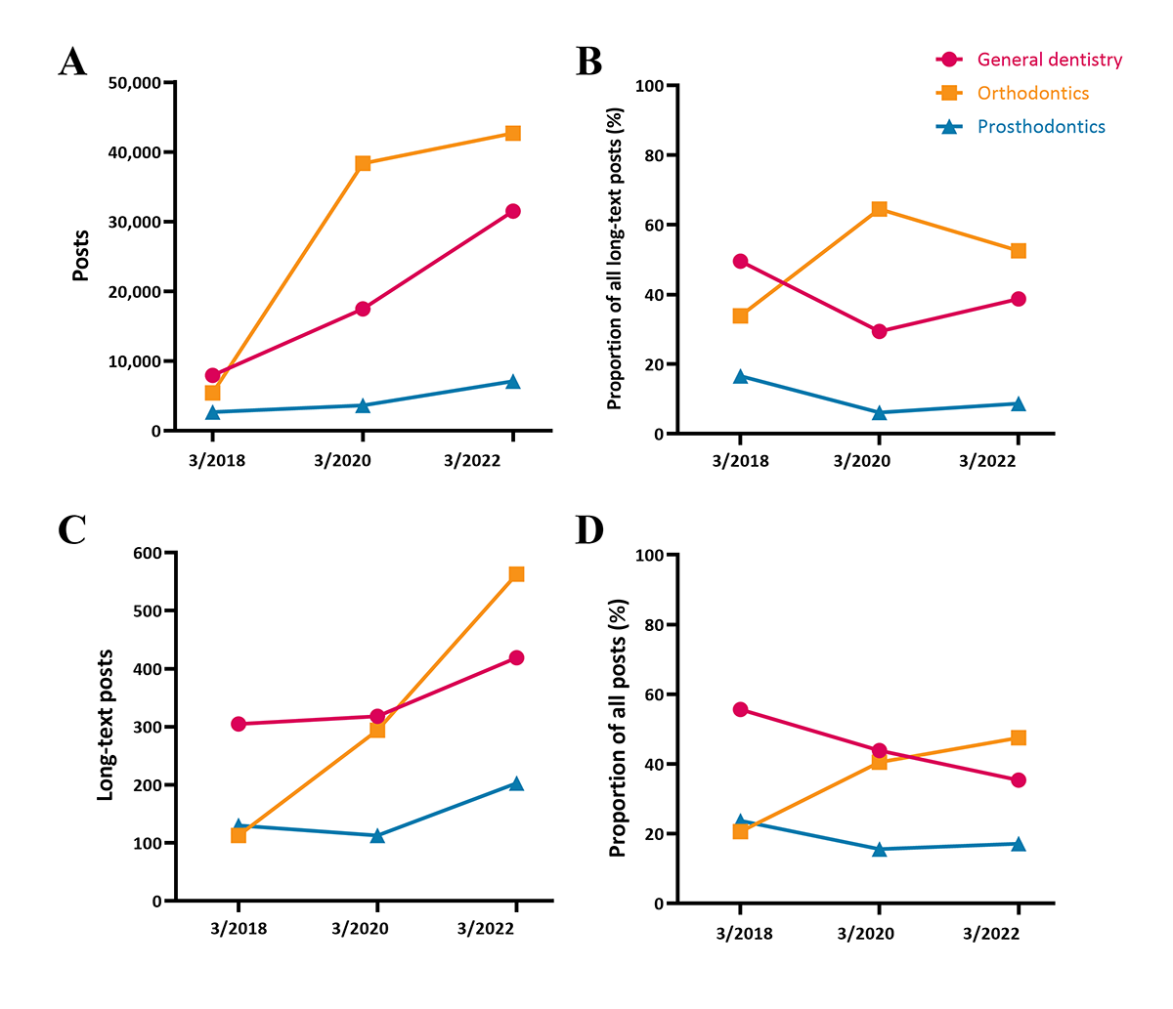
Numbers and proportions of Weibo posts (A, B) and long-text Weibo posts (C, D) on each theme in March 2018, 2020, and 2022.

The research methodology is depicted in Figure 1. According to the search keywords, we retrieved a total of 220,869 Weibo posts. After applying the exclusion criteria, 64,039 (29%) posts were deleted, and the remaining 156,830 (71%) posts were included in the ODB. Among Weibo posts, 2458 (1.6%) were long-text posts. The distribution and proportion of posts and long-text posts for each theme in each observation period are shown in Table S2 in Multimedia Appendix 1. Results showed that over time, there has been significant growth in the content related to dental health care on Weibo, with the number of posts increasing by more than 4 times (Figure 2A). In terms of themes, in 2018, there was a greater proportion of posts and long-text posts discussing general dentistry–related topics, accounting for 49.5% (n=7952) and 55.7% (n=305) of the total, respectively. In 2022, 52.5% (n=42,701) of the posts and 47.5% (n=563) of the long-text posts discussed orthodontics topics (Figure 2D), surpassing the proportion of general dentistry topics. In addition, 543 (0.34%) posts from WeChat and 210 (0.13%) posts from Zhihu were included in the ODB.

**Figure 3:**
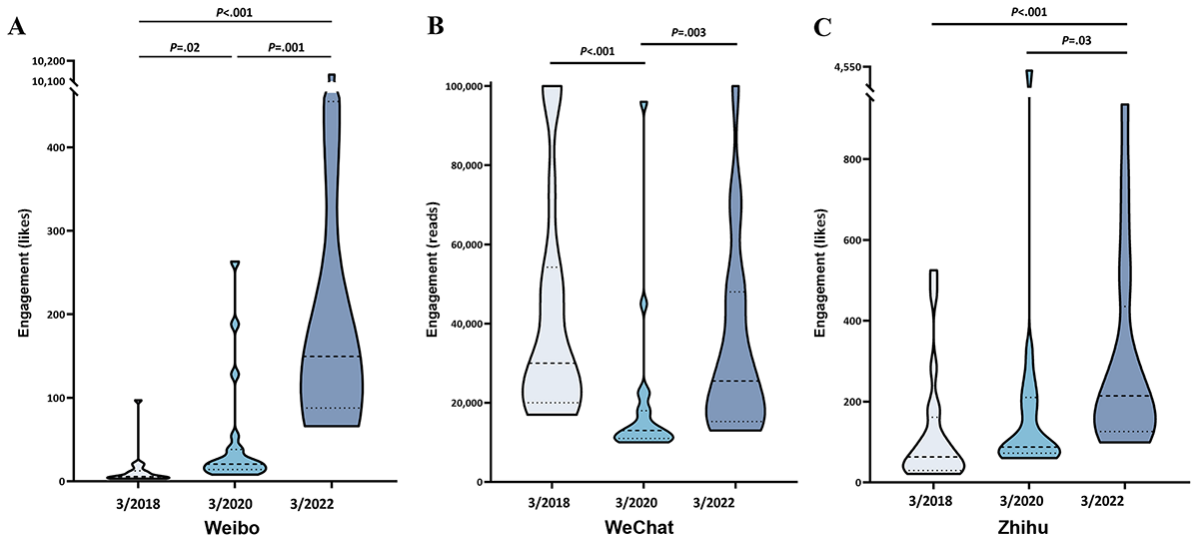
Violin plots of engagement indicators for long-text posts on Weibo (A), WeChat (B), and Zhihu (C) in March 2018, 2020, and 2022.

The ADB comprised 180 most popular long-text posts (n=60, 33.3%, posts from each platform) during the observation period. There were significant differences in the engagement of long-text posts published on Weibo, WeChat, and Zhihu between March 2018, 2020, and 2022 (*P*<.001; Figure 3 and Table S3 in Multimedia Appendix 1). The median (IQR) of likes on Weibo increased from 5.5 (IQR 3.25-12.5) in 2018 to 149.5 (IQR 87.75-454.75) in 2022. Similarly, the median number of likes on Zhihu increased from 63 (IQR 29.25-161.25) in 2018 to 214 (IQR 126-435.5) in 2022. In contrast, the median (IQR) of WeChat reads was 30,000 (IQR 20,000-54,250) in 2018, decreased to 13,000 (IQR 11,000-18,000) in 2020, and then increased to 25,500 (IQR 15,250-48,000) in 2022. Interestingly, 143 (79.4%) long-text posts were written by non–health care professionals, including patients and some medical self-media accounts. In terms of topics, there were 105 (58.3%) posts about personal medical experiences, a number significantly greater than the content of health education and popular science provided by dental health care professionals.

**Figure 4:**
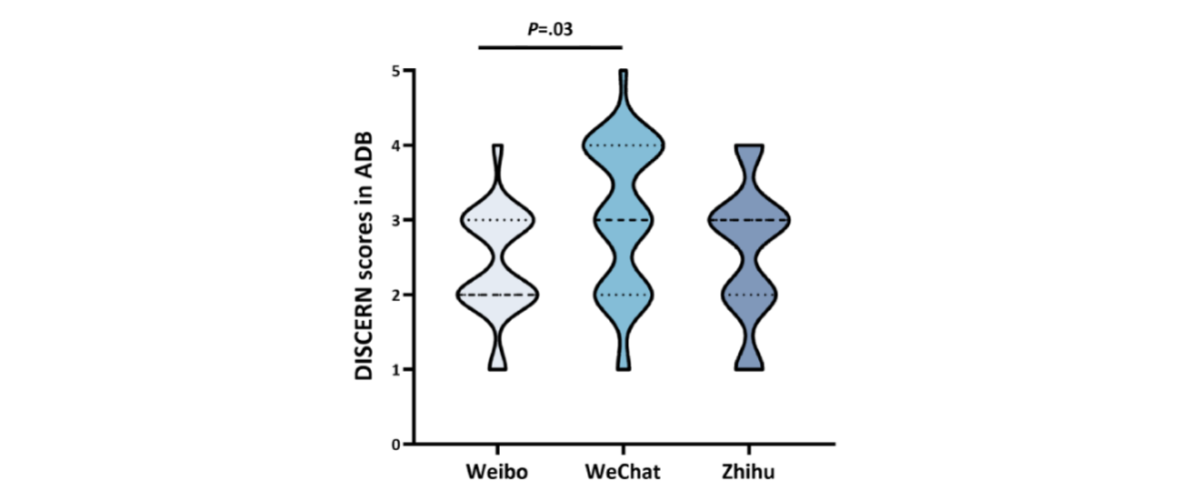
Violin plot of the DISCERN scores of long-text posts on the 3 SMPs in the ADB during the observation period. ADB: analysis database; SMP: social media platform.

In the ADB, the median (IQR) of the DISCERN score for WeChat long-text posts was 3 (IQR 2-4), that for Weibo was 2 (IQR 2-3), and that for Zhihu was 2 (IQR 2-3). There was a significant difference in the DISCERN scores between WeChat and Weibo (*P*=.03; Figure 4 and Table S4 in Multimedia Appendix 1). The scores for each question in the DISCERN questionnaire corresponding to each platform are presented in Table S5 in Multimedia Appendix 1. Among the 180 long-text posts in the ADB, only 37 (20.6%) were authored by dental health care professionals, while the DISCERN scores of these long-text posts on Weibo and WeChat were significantly higher than those of long-text posts written by non–health care professionals. Specifically, for Weibo the median (IQR) of the DISCERN score of health care professionals' long-text posts was 3 (2.5-3.5), while that of non-health care professionals' was 2 (2-3); for WeChat the median (IQR) of the DISCERN score for health care professionals’ long-text posts on WeChat was 4 (IQR 4-4), while that for non–health care professionals was 3 (IQR 2-3). The difference was statistically significant (Weibo *P*=.04; WeChat *P*=.02). Furthermore, there was a significant negative correlation between information quality (DISCERN score) and engagement (Weibo τb=–0.45, *P*=.01; WeChat τb=–0.30, *P*=.02). No similar significant negative correlation was observed for the Zhihu long-text posts.

**Figure 5:**
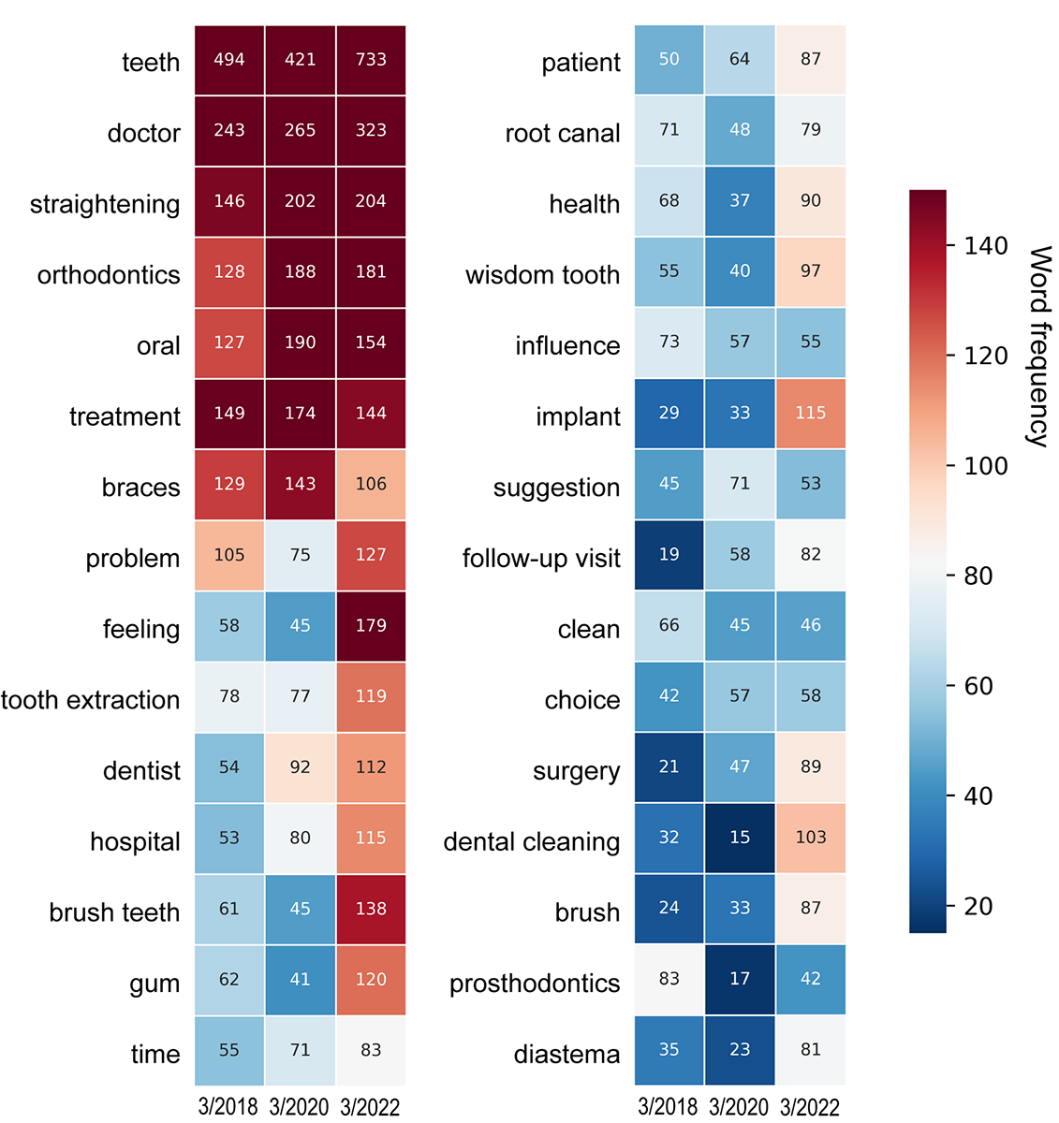
Heatmap of the frequencies of the top 30 words of dental health care information in the ADB in March 2018, 2020, and 2022. Orthodontics and tooth extraction have consistently been the most mentioned terms in Chinese SMPs in regard to dental health care. However, there was a shift from topics such as root canal treatments and metal crown restoration, which were discussed more often in 2018, to topics such as teeth brushing, gingival health, and dental cleaning by 2022. ADB: analysis database; SMP: social media platform.

**Figure 6:**
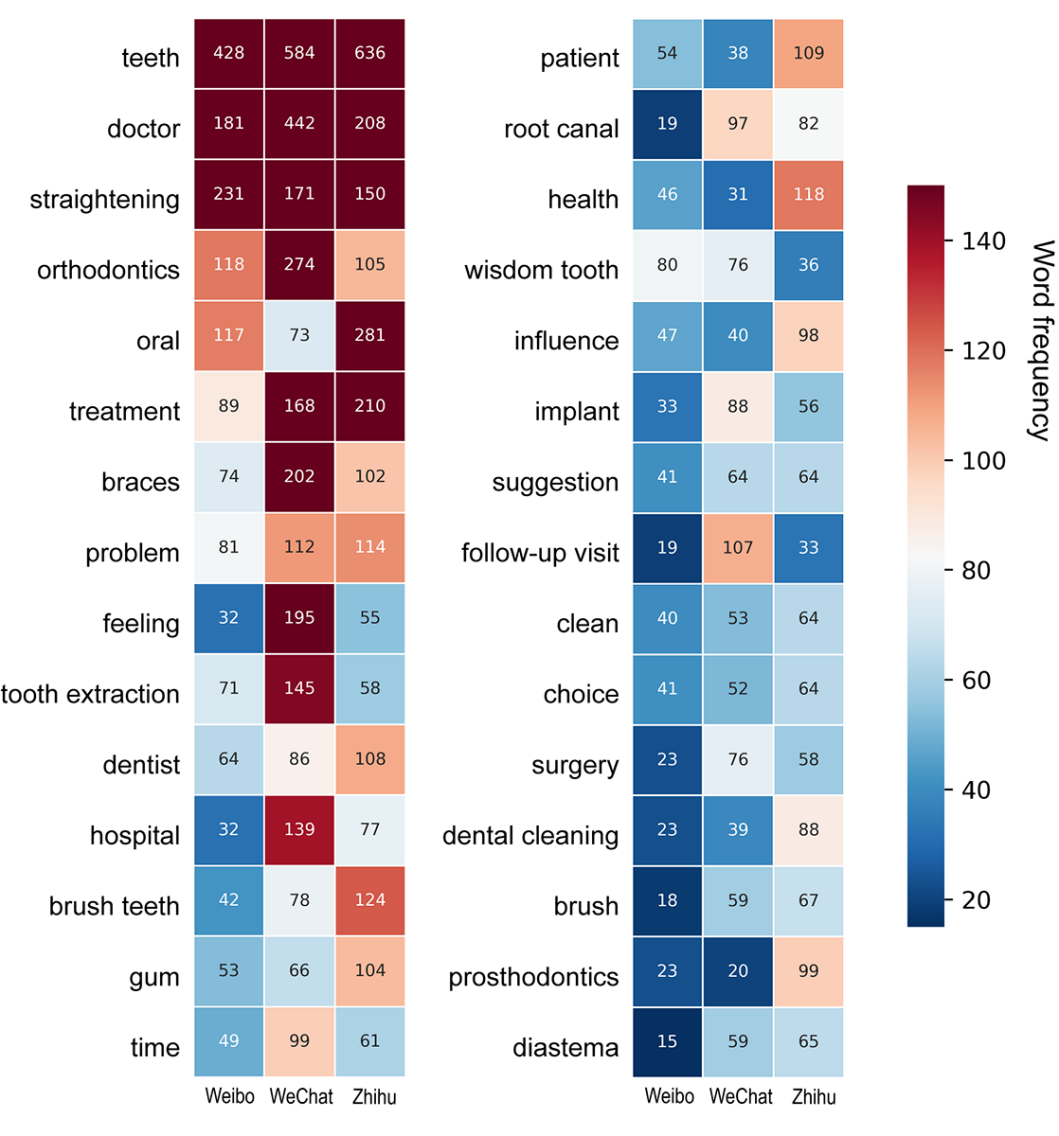
Heatmap of the frequencies of the top 30 words of dental health care information across the 3 SMPs in the ADB. ADB: analysis database; SMP: social media platform.

Text mining analysis of the ADB using NLP tools was visualized as word clouds and heatmaps of the 3 observation periods (Figure 5) and the 3 platforms (Figure 6). For optimal readability, the bilingual word cloud figures are shown in Multimedia Appendices 3 and 4. The frequencies of the top 30 words in each time period and platform are presented in Tables S6 and S7, respectively, in Multimedia Appendix 1. In the word clouds, “teeth” and “doctor” were the most frequently mentioned core keywords. The most prominent terms in the 2018 word cloud were “straightening,” “braces,” “orthodontics,” “prosthodontics,” “tooth extraction,” “root canal,” “health,” and “metal” (crown). In the 2020 word cloud, the most prominent terms were “straightening,” “orthodontics,” “braces,” “tooth extraction,” “invisible,” “deciduous teeth,” “follow-up visit,” and “retention.” In the 2022 word cloud, the most prominent terms were “straightening,” “orthodontics,” “feeling,” “brush teeth,” “gums,” “tooth extraction,” “implant,” and “dental cleaning.” This trajectory suggested that over time, orthodontics and tooth extraction have consistently been the most mentioned terms in Chinese social media in regard to dental health care. However, it could be seen from the word clouds and heatmaps that there was a shift from topics such as root canal treatments and metal crown restoration, which were discussed more often in 2018, to topics such as teeth brushing, gingival health, and dental cleaning by 2022, indicating the concentration on periodontal health and early prevention of dental diseases. There was also an increase in discussions related to dental implantation and orthognathic surgery. The most prominent terms in the Weibo word cloud were “straightening,” “orthodontics,” “wisdom tooth,” “tooth extraction,” and “gum.” The most prominent terms in the WeChat word cloud were “straightening,” “brush teeth,” “health,” “deciduous teeth,” “orthodontics,” and “gum.” In Zhihu, the most prominent terms were “orthodontics,” “braces,” “feeling,” “straightening,” “tooth extraction,” and “follow-up visit.”

## Discussion

### Principal Findings

This study extracted dental health care-related content from Weibo, WeChat, and Zhihu for March 2018, 2020, and 2022 and constructed an ODB. We also constructed an ADB consisting of the most popular long-text posts on each platform. By analyzing the nature, themes, public engagement, information quality, and word frequency, this study tracked the evolution of Chinese social media content related to dental health care. In the field of dentistry, previous studies have investigated SMPs, such as Facebook, Instagram, and YouTube, analyzing topic trends and evaluating the adequacy of these platforms as patient sources of information or education [31-33]. Graf et al [32] explored the nature and potential attitude differences in German orthodontic content on Twitter and Instagram, finding “getting braces” and “getting braces removed” to be the most crucial events for orthodontic patients, and Instagram contained more posts with positive emotions. Yahya et al [34] studied the 63 most viewed videos on YouTube related to miniscrew anchorage and found that the information quality of videos uploaded by dental professionals is not perfect, especially in terms of treatment duration, maintenance, and costs. Similarly, Samur et al [35] reported that the reliability and information quality of content related to facial trauma on SMPs are generally low, highlighting the need for caution when recommending SMPs as a source of facial trauma–related information. To the best of our knowledge, similar systematic research on dental health care information on Chinese SMPs has not been reported in the literature.

Our findings revealed significant growth, by more than 4 times, in Weibo posts concerning dental health care from 2018 to 2022, with the fastest increase observed in the discussion of orthodontics, surpassing general dentistry content since 2020. The long-text posts with the highest engagement on Weibo and Zhihu platforms in the ADB also displayed an upward trend. SMPs plays a progressively significant role in people’s lives [36], and our data confirmed that the amount of dental health care–related content on Chinese SMPs is also steadily increasing. Yang et al [37] reported that Instagram accounts created by oral and maxillofacial surgery residency programs increased exponentially from the period of the 7 months from June to December 2020 compared to the 18 months from December 2018 to May 2020. This indirectly reflected the increasing trend of dental health care information on US SMPs, which is consistent with our results.

In the ADB, which consisted of the most popular content on the 3 SMPs, 79.4% of the long-text posts were written by individuals without a health care background, and 58.3% shared personal medical experiences. This finding was consistent with the study by Samur et al [35], who found that personal experience–based content posted by laypersons receives more interactions. This phenomenon aligns with the theories of cognitive dissonance and selective exposure: people are inclined to consume content that is more similar and relatable to them [38,39]. It is worth noting that long-text posts authored by health care professionals received significantly higher DISCERN scores, indicating superior information quality. Nonetheless, these long-text posts were not rewarded with the same level of engagement as those written by nonprofessionals. Furthermore, there was a negative correlation between the DISCERN scores and engagement observed on Weibo and WeChat, suggesting that high-quality information may not generate a larger audience. Similar trends were observed in the study by Hegarty et al [40], who found that the most viewed YouTube videos are less helpful. However, a study by Kovalski et al [41] on oral leukoplakia–related content showed that more reliable videos of higher quality receive more likes and have higher viewing rates and interaction indices. Studies indicate that misinformation often features sensational headlines that are easy to understand without deep engagement or critical thinking. These posts quickly capture attention, eliciting strong emotional responses that prompt users to like, comment, and share [9,42]. The positive feedback loop of social media algorithms amplifies the spread of low-quality content, indirectly suppressing accurate, evidence-based, high-quality information and creating information silos [43].

Peek et al’s [44] guidelines for mental health education and advocacy noted that using language that is more accessible to the public instead of medical jargon can make popular science even more popular. However, in the study by Yahya et al [34] on 31 videos uploaded by dental professionals about miniscrew anchorage on YouTube, only 2 videos avoided using technical terms. The remaining videos all used them, with 80.7% failing to provide explanations. The excessive use of technical terms, obscure and complicated principles, and stagnant formatting in health education materials may meet the requirements of the DISCERN questionnaire and thus can elicit high information quality scores. However, several studies have revealed that this may result in a loss of readers’ interest and psychological resonance [35,39]. Conversely, using psychologically assisted writing techniques that foster affinity may prove more effective in attracting readers and achieving better health education outcomes. To effectively disseminate health care knowledge, dental professionals should improve writing methodologies, while ensuring the accuracy and high quality of the evidence-based information conveyed. For instance, incorporating patient-centered medical experiences and visual materials can attract more readers and stimulate discussions. This could ultimately make a greater impact and benefit a larger audience. In addition to health care professionals, governments and health departments should take measures to promote the dissemination of high-quality health information online, while enhancing the public’s critical-thinking skills to discern true from false information and make informed decisions [9,13]. Technology platforms should transparentize and optimize recommendation algorithms and regulate the quality of health [9,42].

Oliveira et al [45] performed 2 searches on Twitter using the keywords “dentist” and “teeth” and generated a word cloud based on the collected tweets, finding that the most commonly used terms are “third molar” and “orthodontic appliance.” On this basis, they determined that the most common dental needs during the COVID-19 pandemic were pain, urgencies, and orthodontic follow-ups. Graf et al [32] showcased word clouds of orthodontically posts with different sentiments. Positive posts revolved around the effectiveness of orthodontic treatment and the excitement of wearing or removing braces, while negative posts covered complaints about appointments, waiting times, pain, and side effects during orthodontic treatment. In this study, word clouds were generated for 3 SMPs during the observation period. We found that “teeth” and “doctors” are consistently the core subjects in the field of dental health care on Chinese SMPs. In line with the findings of Oliveira et al [45], orthodontics and tooth extraction have been the most discussed topics across different years and platforms, suggesting that the most prevalent keywords on Chinese SMPs are similar to those in other languages. Moreover, there has been a noticeable shift from topics such as endodontic treatment or dental crown restoration in 2018 to a stronger emphasis on topics such as periodontal maintenance and early prevention by 2022. This is indicated by the increased discussions around teeth brushing, gingival health, and dental cleaning. Additionally, topics such as dental implantation and orthognathic surgery have gradually gained popularity in the word clouds, suggesting that concepts in these areas are becoming more universally accepted by the public. Patients interest has gradually evolved from basic dental treatments to functional dentofacial aesthetics and preventative care.

This study demonstrates the increase in the quantity and engagement of dental health information on Chinese social media from 2018 to 2022, emphasizing the importance of offering high-quality information online in the digital age. Additionally, we explored changes in public interest in dental topics, providing insights into the evolving awareness of patients. This highlights the need for dental health care providers to supplement evidence-based information, particularly on topics of interest to the general public. They should take measures to improve the popularity of online scientific materials and mitigate the impact of low-quality information.

### Limitations

This study has a few limitations. First, there are constrained capabilities of NLP tools when evaluating complex indicators, such as DISCERN scores, across large datasets, as well as identifying meaningless content, such as bot-generated text. Consequently, this task was performed manually as a substitute, which inevitably restricted the scale of relevant data analysis, necessitating the use of sampling methods rather than analyzing the entire dataset. Second, platforms such as WeChat and Zhihu do not provide a straightforward way to access comprehensive data, prompting us to use alternative strategies for collecting a representative sample. We also note that including data from 2019 and 2021 could have resulted in more continuous and reliable sampling points. Third, this study was retrospective in design, while, given the dynamic nature of social media, some users may have hidden or deleted previously published posts, potentially introducing bias into the findings. Finally, expanding the scope of this study to include data from additional sources, such as government agencies or dental associations, would facilitate a comparative analysis. Future research could focus on these aspects.

### Conclusion

During 2018-2022, despite the increase in the dissemination and evolution of public interest in dental health care information on Chinese social media, the quality of the most popular long-text posts was rated as moderate or low, which may mislead patients and the public. These findings could yield insights for dental practitioners, investigators, and educators into patients’ evolving perceptions and interests in the era of social media. We also emphasize the importance of enhancing the provision of high-quality and popular health information on Chinese SMPs.

## Appendix

Multimedia Appendix 1 Chinese keywords for searching posts and their corresponding English translation; distribution of the number and proportion of Weibo and long-text posts on different themes in the ODB; engagement of long-text posts on Weibo, WeChat, and Zhihu in the ADB; DISCERN scores of long-text posts on the 3 SMPs in the ADB during the observation period; DISCERN scores of each question of long-text posts on the 3 SMPs in the ADB during the observation period; frequencies of the top 30 words in the ADB in March 2018, 2020, and 2022 and on Weibo, WeChat, and Zhihu. ADB: analysis database; ODB: original database; SMP: social media platform.

Multimedia Appendix 2 DISCERN health information quality assessment questionnaire.

Multimedia Appendix 3 Word clouds in English.

Multimedia Appendix 4 Word clouds in Chinese.

## Abbreviations

ADB: analysis database
AI: artificial intelligence
NLP: natural language processing
ODB: social media original database
SMP: social media platform

## Data Availability

The databases generated and analyzed in this work are available from the corresponding author upon reasonable request.
