# Supplementary material for: Evolutionary Trend of Dental Health Care Information on Chinese Social Media Platforms During 2018-2022: Retrospective Observational Study
Source: JMIR Infodemiology. 2025 Apr 10;5:e55065. doi: 10.2196/55065 (PMC12022532; doi:10.2196/55065)
Supplement: Multimedia Appendix 1 [file infodemiology_v5i1e55065_app1.docx]

**Table S1. Chinese keywords for searching the posts and the corresponding English translation**

| Chinese keywords | English keywords(translated) |
| --- | --- |
| 补牙 | dental fillings |
| 洗牙 | dental cleaning |
| 拔牙 | tooth extraction |
| 根管治疗 | root canal treatment |
| 牙齿美白 | teeth whitening |
| 正畸 | orthodontics |
| 牙齿矫正 | teeth straightening |
| 牙齿矫治 | orthodontic treatment |
| 牙套 | braces |
| 牙箍 | dental braces |
| 箍牙 | get brace |
| 保持器 | retainer |
| 牙冠 | dental crown |
| 嵌体 | overlay |
| 瓷牙 | porcelain tooth |
| 种牙 | dental implant |
| 种植牙 | implanted tooth |
| 牙贴面 | tooth veneer |
| 瓷贴面 | porcelain veneer |

**Table S2. Distribution of number and proportion of Weibo posts and long-text posts on different themes in ODB.**

| Theme | 3/2018 | | | | 3/2020 | | | | | | 3/2022 | | | |
| --- | --- | --- | --- | --- | --- | --- | --- | --- | --- | --- | --- | --- | --- | --- |
|  | Posts | Prop. (%) | Long-text posts | Prop. (%) |  | Posts | Prop.  (%) | Long-text posts | Prop.  (%) |  | Posts | Prop.  (%) | Long-text posts | Prop.  (%) |
| General dentistry | 7952 | 49.53 | 305 | 55.66 |  | 17471 | 29.37 | 318 | 43.86 |  | 31501 | 38.75 | 419 | 35.36 |
| Orthodontics | 5441 | 33.89 | 113 | 20.62 |  | 38366 | 64.51 | 294 | 40.55 |  | 42701 | 52.52 | 563 | 47.51 |
| Prosthodontics | 2663 | 16.58 | 130 | 23.72 |  | 3639 | 6.12 | 113 | 15.59 |  | 7096 | 8.73 | 203 | 17.13 |
| Total | 16056 |  | 548 |  |  | 59476 |  | 725 |  |  | 81298 |  | 1185 |  |

Prop., proportion.

**Table S3. Engagement of long-text posts on Weibo, WeChat, and Zhihu in ADB.**

|  |  |  | “Likes” or reads |  | K-W test (H, *P* value) | |
| --- | --- | --- | --- | --- | --- | --- |
|  |  |  | Median (P25-P75） |  |  |  |
| Weibo |  |  |  |  |  |  |
|  | 3/2018 |  | 5.5 (3.25-12.5) |  | 39.98, <.001 | |
|  | 3/2020 |  | 20.5 (14-38) |  |  |  |
|  | 3/2022 |  | 149.5(87.75-454.75) |  |  |  |
| WeChat |  |  |  |  |  |  |
|  | 3/2018 |  | 30000 (20000-54250) |  | 20.81, <.001 | |
|  | 3/2020 |  | 13000 (11000-18000) |  |  |  |
|  | 3/2022 |  | 25500 (15250-48000) |  |  |  |
| Zhihu |  |  |  |  |  |  |
|  | 3/2018 |  | 63 (29.25-161.25) |  | 17.09, <.001 | |
|  | 3/2020 |  | 87.5 (72.25-210.25) |  |  |  |
|  | 3/2022 |  | 214 (126-435.5) |  |  |  |

P25, 25^th^ percentile; P75, 75^th^ percentile; K-W test, Kruskal‒Wallis test.

**Table S4. DISCERN scores of long-text posts on the three SMPs in ADB during the observation period.**

|  | DISCERN scores | K-W test (H, *P* value) |
| --- | --- | --- |
|  | Median (P25-P75) |  |
| Weibo | 2 (2-3) | 6.94, .03 |
| WeChat | 3 (2-4) |  |
| Zhihu | 2 (2-3) |  |

P25, 25th percentile; P75, 75th percentile; K-W test, Kruskal‒Wallis test

**Table S5. DISCERN scores of each question of long-text posts on the three SMPs in ADB during the observation period.**

| Number | DISCERN scores | | |
| --- | --- | --- | --- |
|  | Median (P25-P75) | | |
|  | Weibo | WeChat | Zhihu |
| 1 | 5 (5-5) | 5 (5-5) | 5 (5-5) |
| 2 | 5 (4-5) | 5 (4-5) | 5 (4-5) |
| 3 | 5 (5-5) | 5 (5-5) | 5 (5-5) |
| 4 | 1 (1-2) | 3 (3-4) | 2 (1-3) |
| 5 | 2 (2-2) | 3 (2-4) | 2 (2-3) |
| 6 | 3 (2-3) | 3 (3-3.75) | 3 (3-3) |
| 7 | 1 (1-2.75) | 3.5 (3-5) | 3 (2-4) |
| 8 | 1 (1-2.75) | 4 (3-4) | 2 (2-3.75) |
| 9 | 3 (3-4) | 4 (4-5) | 3 (3-4) |
| 10 | 3 (3-4) | 4 (3-5) | 4 (3-5) |
| 11 | 2 (2-3) | 3 (2-4) | 2.5 (2-3) |
| 12 | 3 (3-4) | 3.5 (2-4) | 3 (2-4) |
| 13 | 3 (3-4) | 3 (3-4) | 3 (2-3) |
| 14 | 3 (2-3) | 3 (3-4) | 3 (2-4) |
| 15 | 1 (1-1) | 1 (1-3.75) | 1 (1-1.75) |

P25, 25th percentile; P75, 75th percentile

**Table S6. The frequencies of the top 30 words in ADB in March 2018, 2020, and 2022.**

| Rank | 3/2018 | Frequency | 3/2020 | Frequency | 3/2022 | Frequency |
| --- | --- | --- | --- | --- | --- | --- |
| 1 | teeth | 494 | teeth | 421 | teeth | 733 |
| 2 | doctor | 243 | doctor | 265 | doctor | 323 |
| 3 | treatment | 149 | straightening | 202 | straightening | 204 |
| 4 | straightening | 146 | oral | 190 | orthodontics | 181 |
| 5 | braces | 129 | orthodontics | 188 | feeling | 179 |
| 6 | orthodontics | 128 | treatment | 174 | oral | 154 |
| 7 | oral | 127 | braces | 143 | treatment | 144 |
| 8 | problem | 105 | dentist | 92 | brush teeth | 138 |
| 9 | prosthodontics | 83 | hospital | 80 | problem | 127 |
| 10 | tooth extraction | 78 | tooth extraction | 77 | gum | 120 |
| 11 | influence | 73 | problem | 75 | tooth extraction | 119 |
| 12 | root canal | 71 | suggestion | 71 | hospital | 115 |
| 13 | health | 68 | time | 71 | implant | 115 |
| 14 | metal | 67 | invisible | 69 | dentist | 112 |
| 15 | clean | 66 | deciduous teeth | 64 | braces | 106 |
| 16 | gum | 62 | patient | 64 | dental cleaning | 103 |
| 17 | brush teeth | 61 | follow-up visit | 58 | wisdom tooth | 97 |
| 18 | feeling | 58 | influence | 57 | health | 90 |
| 19 | wisdom tooth | 55 | choice | 57 | surgery | 89 |
| 20 | time | 55 | retention | 57 | patient | 87 |
| 21 | dentist | 54 | face | 53 | brush | 87 |
| 22 | hospital | 53 | root canal | 48 | time | 83 |
| 23 | dental filling | 52 | children | 47 | follow-up visit | 82 |
| 24 | patient | 50 | surgery | 47 | diastema | 81 |
| 25 | suggestion | 45 | brush teeth | 45 | root canal | 79 |
| 26 | metal-porcelain crown | 45 | clean | 45 | toothpaste | 76 |
| 27 | floss | 44 | feeling | 45 | occlusion | 68 |
| 28 | effect | 43 | effect | 45 | toothbrush | 66 |
| 29 | retention | 43 | technology | 44 | choice | 58 |
| 30 | pain | 43 | floss | 42 | children | 56 |

**Table S7. The frequencies of the top 30 words in ADB on Weibo, WeChat, and Zhihu.**

| Rank | Weibo | Frequency | WeChat | Frequency | Zhihu | Frequency |
| --- | --- | --- | --- | --- | --- | --- |
| 1 | teeth | 428 | teeth | 636 | teeth | 584 |
| 2 | straightening | 231 | oral | 281 | doctor | 442 |
| 3 | doctor | 181 | treatment | 210 | orthodontics | 274 |
| 4 | orthodontics | 118 | doctor | 208 | braces | 202 |
| 5 | oral | 117 | straightening | 150 | feeling | 195 |
| 6 | treatment | 89 | brush teeth | 124 | straightening | 171 |
| 7 | problem | 81 | health | 118 | treatment | 168 |
| 8 | wisdom tooth | 80 | problem | 114 | tooth extraction | 145 |
| 9 | braces | 74 | deciduous teeth | 114 | hospital | 139 |
| 10 | tooth extraction | 71 | patient | 109 | problem | 112 |
| 11 | dentist | 64 | dentist | 108 | follow-up visit | 107 |
| 12 | patient | 54 | orthodontics | 105 | time | 99 |
| 13 | gum | 53 | gum | 104 | root canal | 97 |
| 14 | time | 49 | braces | 102 | implant | 88 |
| 15 | influence | 47 | prosthodontics | 99 | dentist | 86 |
| 16 | health | 46 | influence | 98 | face | 83 |
| 17 | brush teeth | 42 | dental cleaning | 88 | brush teeth | 78 |
| 18 | choice | 41 | metal | 84 | wisdom tooth | 76 |
| 19 | suggestion | 41 | root canal | 82 | surgery | 76 |
| 20 | clean | 40 | children | 82 | oral | 73 |
| 21 | metal-porcelain crown | 37 | hospital | 77 | gum | 66 |
| 22 | anesthetics | 35 | material | 77 | suggestion | 64 |
| 23 | children | 34 | metal-porcelain crown | 72 | occlusion | 63 |
| 24 | effect | 34 | toothbrush | 71 | change | 63 |
| 25 | implant | 33 | examination | 71 | brush | 59 |
| 26 | feeling | 32 | brush | 67 | diastema | 59 |
| 27 | hospital | 32 | diastema | 65 | toothpaste | 58 |
| 28 | retention | 32 | suggestion | 64 | retention | 57 |
| 29 | examination | 30 | clean | 64 | pain | 57 |
| 30 | plan | 30 | choice | 64 | friend | 56 |
