# Supplementary material for: Evolutionary Trend of Dental Health Care Information on Chinese Social Media Platforms During 2018-2022: Retrospective Observational Study
Source: JMIR Infodemiology. 2025 Apr 10;5:e55065. doi: 10.2196/55065 (PMC12022532; doi:10.2196/55065)
Supplement: Multimedia Appendix 2 [file infodemiology_v5i1e55065_app2.docx]

DISCERN health information quality assessment questionnaire.

| Questionnaire section and items | | | Likert scale score | | | | |
| --- | --- | --- | --- | --- | --- | --- | --- |
| **Is the publication reliable?** | | | No | Partially | | | Yes |
|  | 1 | Are the aims clear? | 1 | 2 | 3 | 4 | 5 |
|  | 2 | Does it achieve its aims? | 1 | 2 | 3 | 4 | 5 |
|  | 3 | Is it relevant? | 1 | 2 | 3 | 4 | 5 |
|  | 4 | Is it clear what sources were used to compile the publication? | 1 | 2 | 3 | 4 | 5 |
|  | 5 | Is it clear when the information used or reported was produced? | 1 | 2 | 3 | 4 | 5 |
|  | 6 | Is it balanced and unbiased? | 1 | 2 | 3 | 4 | 5 |
|  | 7 | Does it provide details of additional sources of support and information? | 1 | 2 | 3 | 4 | 5 |
|  | 8 | Does it refer to areas of uncertainty? | 1 | 2 | 3 | 4 | 5 |
| **How good is the quality of information on treatment choices?** | | | No | Partially | | | Yes |
|  | 9 | Does it describe how each treatment works? | 1 | 2 | 3 | 4 | 5 |
|  | 10 | Does it describe the benefits of each treatment? | 1 | 2 | 3 | 4 | 5 |
|  | 11 | Does it describe the risks of each treatment? | 1 | 2 | 3 | 4 | 5 |
|  | 12 | Does it describe what would happen if no treatment is used? | 1 | 2 | 3 | 4 | 5 |
|  | 13 | Does it describe how the treatment choices affect overall quality of life? | 1 | 2 | 3 | 4 | 5 |
|  | 14 | Is it clear that there may be more than one possible treatment choice? | 1 | 2 | 3 | 4 | 5 |
|  | 15 | Does it provide support for shared decision-making? | 1 | 2 | 3 | 4 | 5 |
| **Overall rating of the publication** | | | Low | Moderate | | | High |
|  | | | | | | | |
|  | 16 | Based on the answers to the above questions, rate the overall quality. | 1 | 2 | 3 | 4 | 5 |
