# Supplementary figures and images for: Evolutionary Trend of Dental Health Care Information on Chinese Social Media Platforms During 2018-2022: Retrospective Observational Study
Source: JMIR Infodemiology. 2025 Apr 10;5:e55065. doi: 10.2196/55065 (PMC12022532; doi:10.2196/55065)

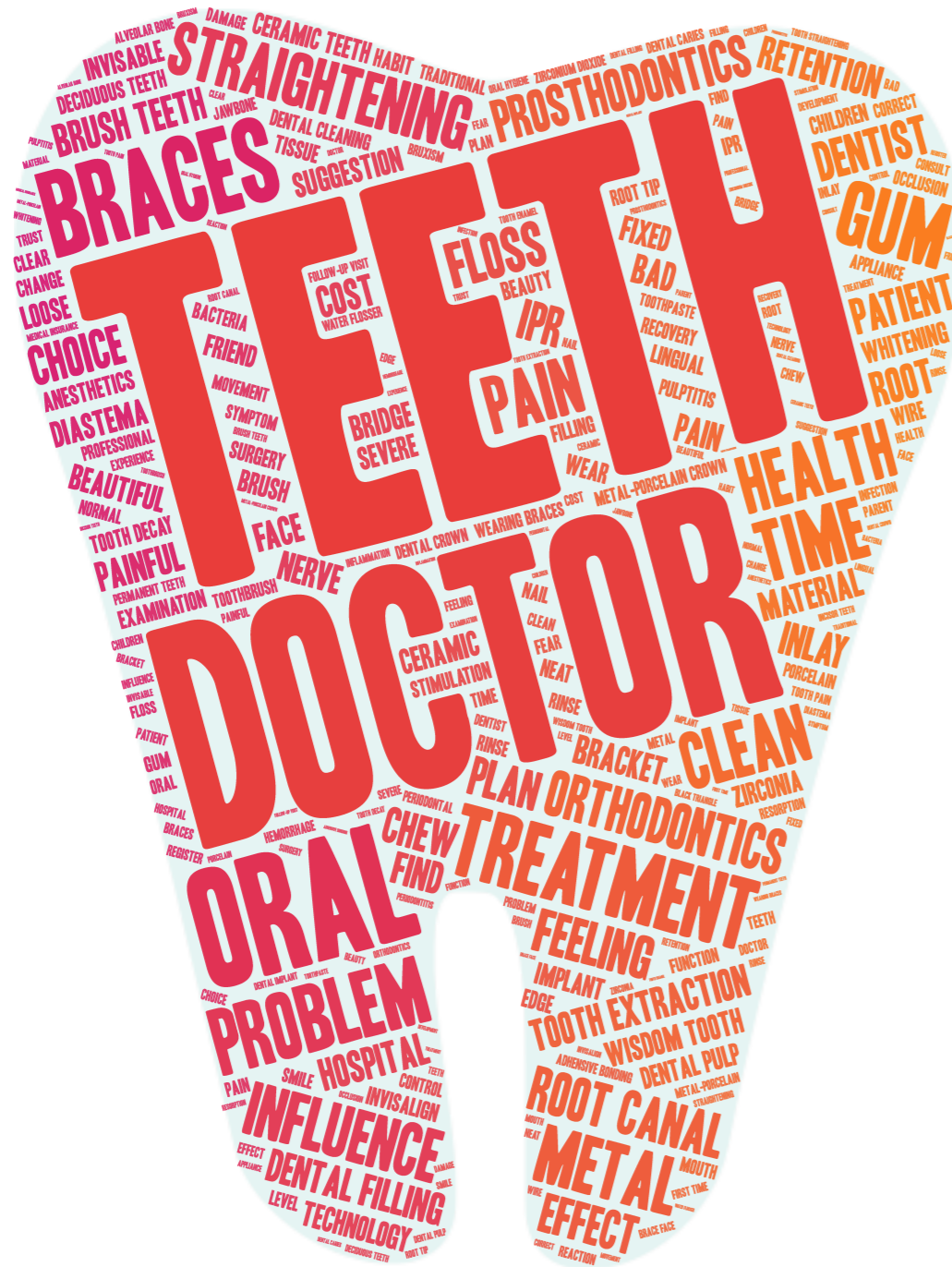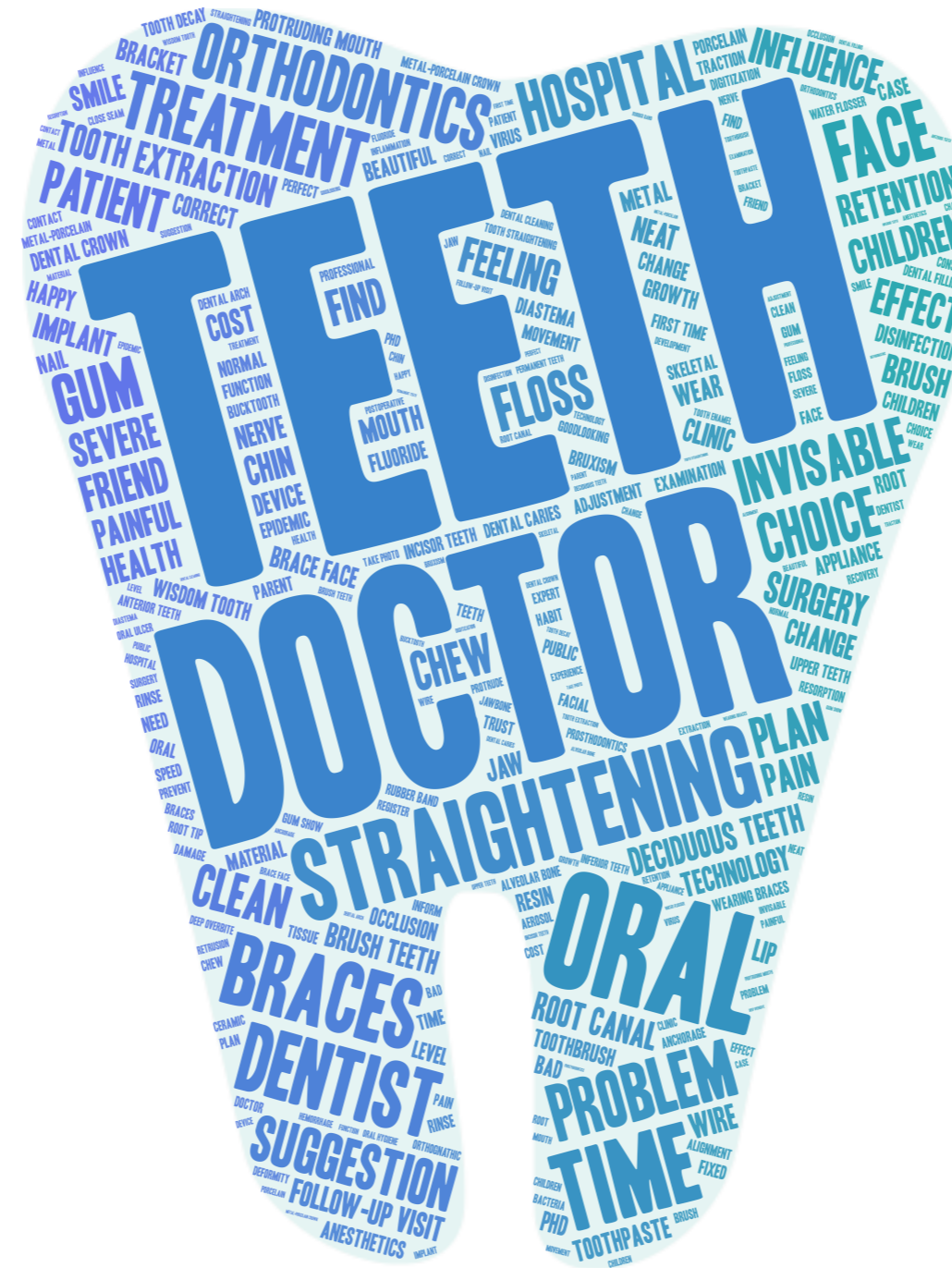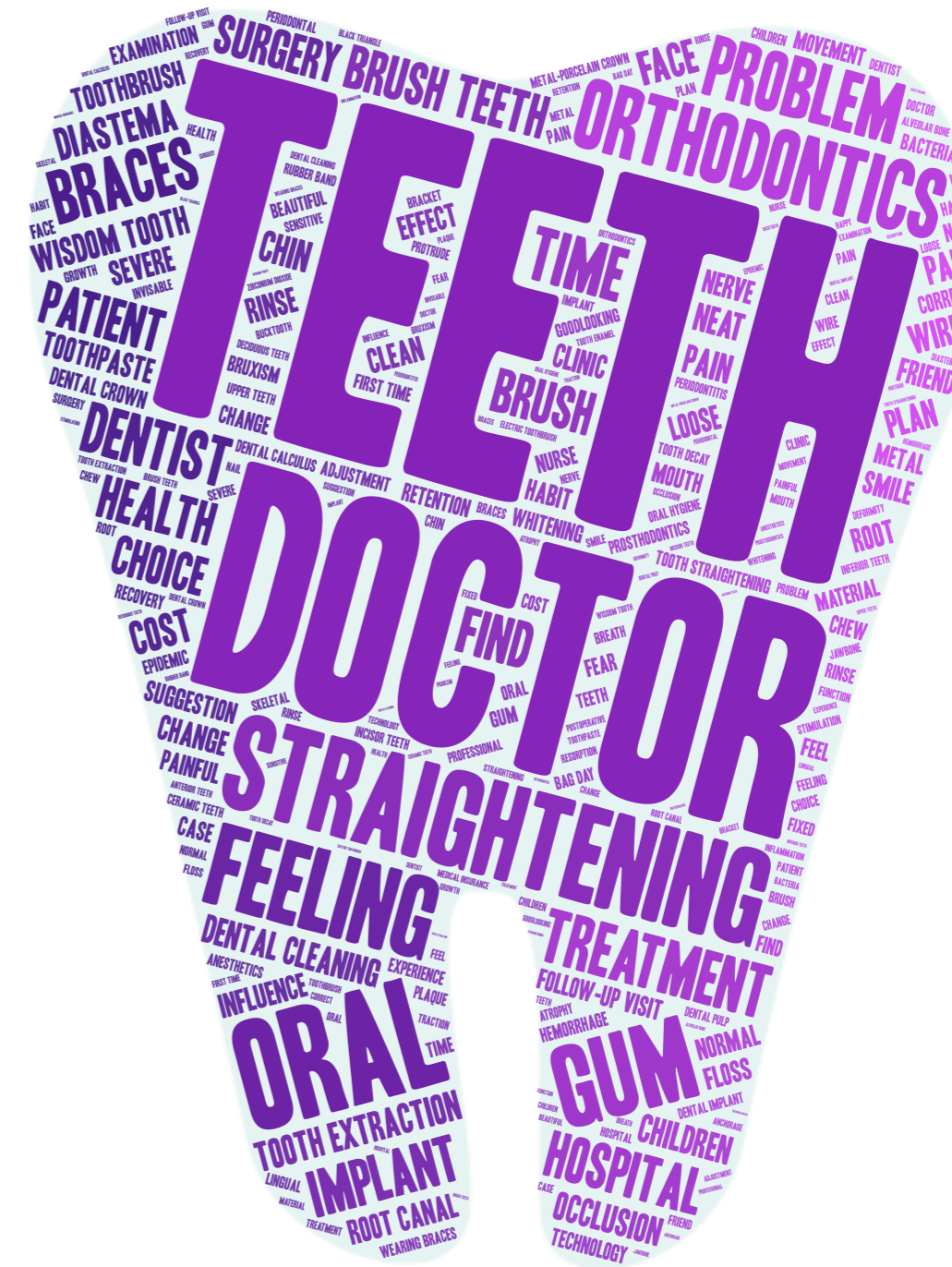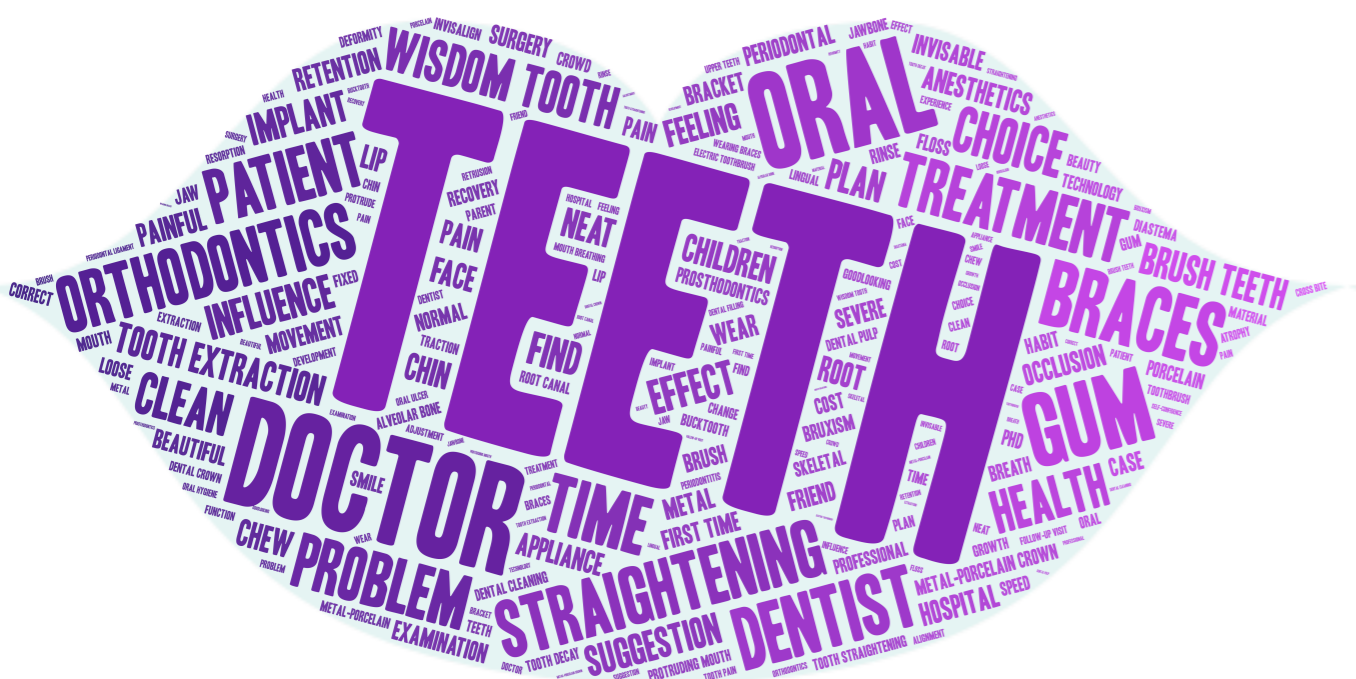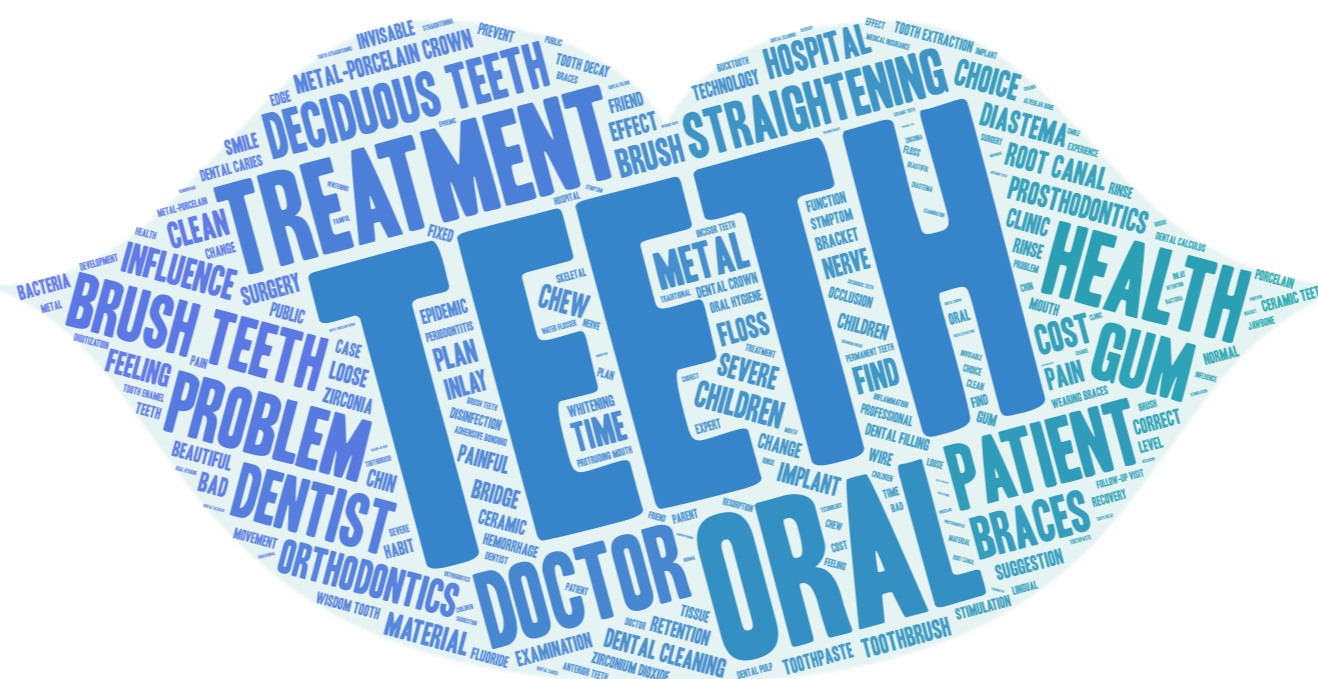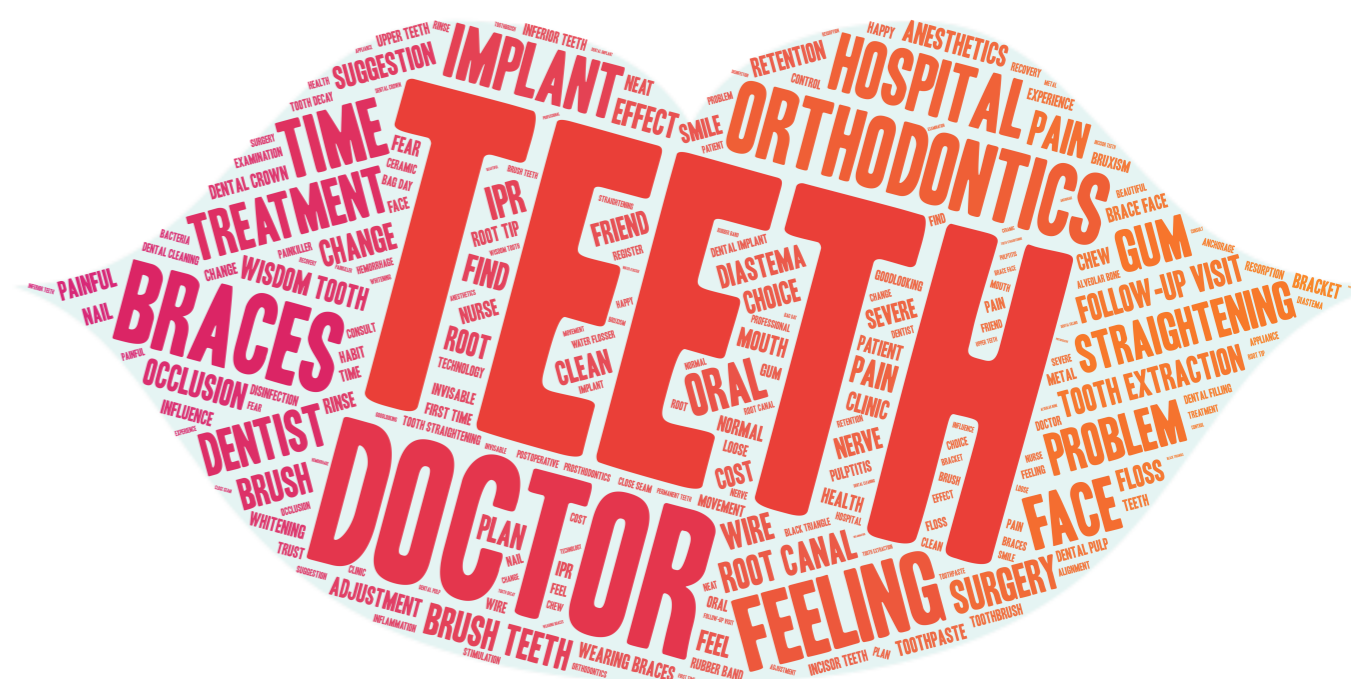

Supplement: Multimedia Appendix 3 [file infodemiology_v5i1e55065_app3.pdf]
