# Supplementary material for: Evolutionary Trend of Dental Health Care Information on Chinese Social Media Platforms During 2018-2022: Retrospective Observational Study
Source: JMIR Infodemiology. 2025 Apr 10;5:e55065. doi: 10.2196/55065 (PMC12022532; doi:10.2196/55065)
Supplement: Multimedia Appendix 4 [file infodemiology_v5i1e55065_app4.pdf]

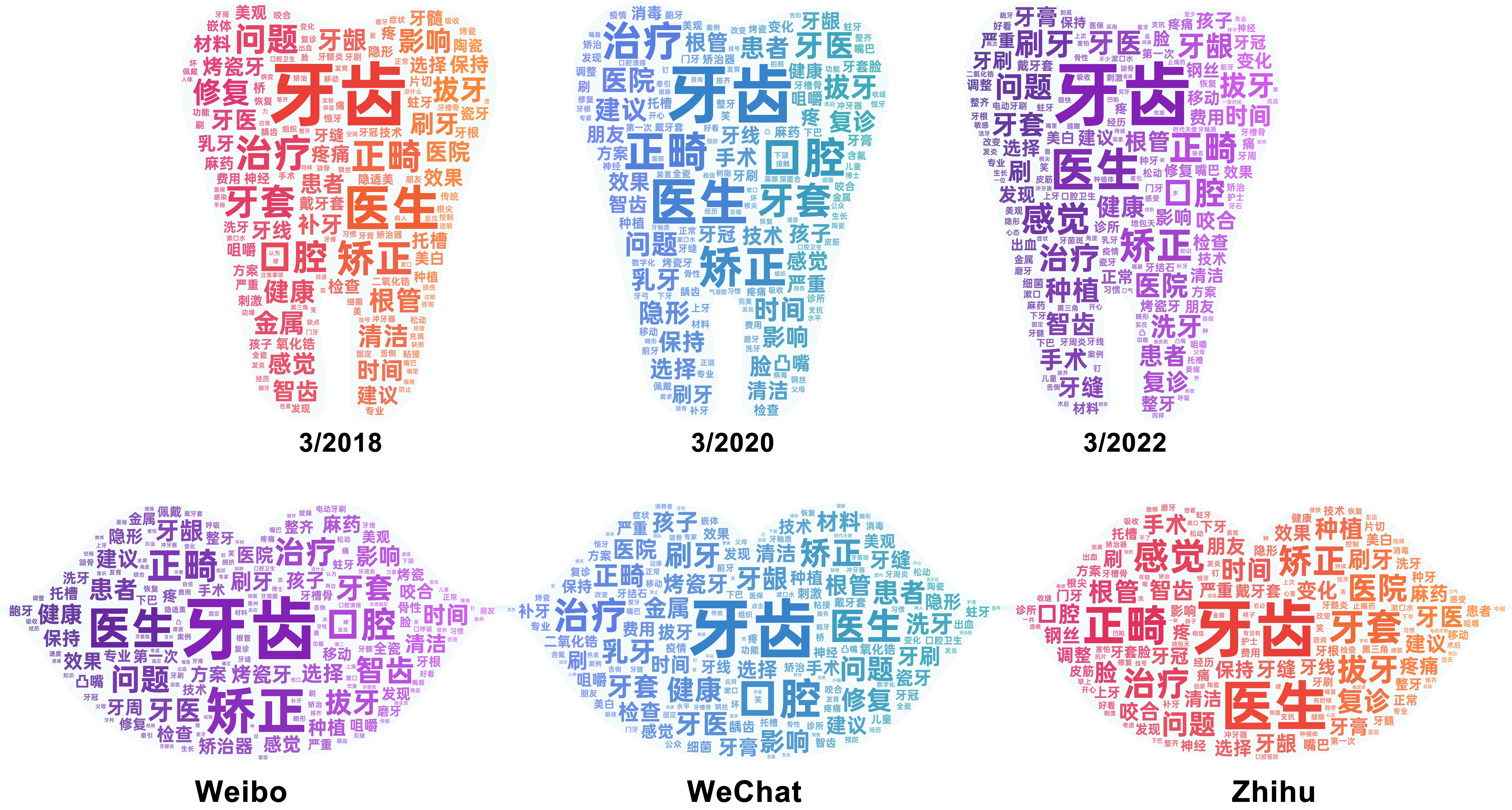
3/2018
3/2020
3/2022
Weibo
WeChat
Zhihu
